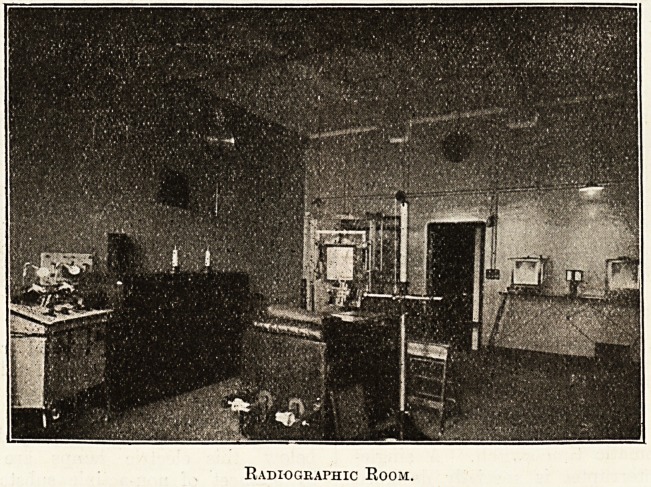# Radio-Therapeutic Department at New King's

**Published:** 1914-10-17

**Authors:** 


### RADIO-THERAPEUTIC DEPARTMENT AT NEW KING'S.

### Official Account of its Plan and Equipment.

The new King's College Hospital has one of the most up-to-date radio-therapeutic departments in England. Almost an entire floor of the out-patient department is utilised; it is divided up into rooms opening into a corridor which divides the radiographic on one side from the therapeutic department on the other. The radiographic room, with a floor space of 24 feet by 26 feet, contains a 20-inch spark coil, with a five-way subdivided primary especially designed to meet the requirements of every kind of work.

This apparatus is sufficiently powerful to

Treatment Cubicle.

take a radiograph in one-hundredth part of a second. By the use of various switches the apparatus can be worked by a multiple-point electrolytic interrupter, for exposures varying from one-tenth of a second to ten seconds. This is controlled by an automatic time-switch. A single-point electrolytic interrupter is specially designed so that the depth of its platinum point in the electrolic can be regulated by a resistance at the switch-table. A " Sanax " interrupter is also used in conjunction with the apparatus.

In the centre of the ceiling is mounted a three-way high-tension switch controlled by hanging silk cords, enabling the operator instantly to connect or disconnect the upright stand that is used principally for bismuth meals and chest examinations. It controls the Albers-Schonberg couch, also the special couch (designed by the superintendent) that is seen in the centre of the picture. The whole under-framework forms a complete lead-lined protective cabinet, within which is the $x$-ray tube that can be adjusted vertically, horizontally, or transversely, quite easily. Above the couch is a tube-stand used for compression.

Connected with the radiographic room by double doors are two well-equipped dark rooms—the outer,

Diathermy Room.

containing an enlarging and reducing camera, illuminated by two powerful arc lamps. A special feature of the inner dark room is an automatic developing machine, driven by an electric motor. The bottom of the developing dish is of glass; below this electric lamps are placed, covered by a sheet of non-actinic substance, so that the image on the plate can be examined during development.

The dark room is fitted with red safety lamps. A viewing box for examining wet negatives is situated between the hypo and washing tanks. There is also a smaller tank used for rinsing. All these three tanks are of pine lined with lead, and are fitted with syphons as well as the ordinary plugs, thus enabling small particles to be carried away from the bottom.

A small operating-room is provided for diathermy, coagulation, high-frequency, galvanism, and faradism.

The photographic studio has a whole-plate reflex camera, a suitable background, two quartz mercury lamps used for illumination in dull weather, also for treatment of lupus, rodent ulcer, and other skin diseases.

Photographic Studio.

The same apparatus can be used for "cold" cautery or coagulation. One pole is connected with a needle and the other to a pad strapped to the patient; a small spark gap, about 5 mm. long, discharges when the needle is brought close to the body. A rotary converter is used for changing the current from 110 volts into alternating current for this purpose. Six cubicles are provided for $x$-ray treatment. The walls and doors of these are lined with lead and have lead-glass windows. The apparatus in each cubicle is controlled outside in a corridor which runs the length of the cubicles.

The consulting-room is well equipped with viewing boxes, one specially designed to take six fifteen by twelve negatives. This, however, can be altered to take any-sized negatives. There is a Wheatstone stereoscope and a McKenzie-Davidson localiser for locating foreign bodies in the eye. Also a safe containing 60 milligrammes of radium, used for treatment of rodent ulcers, etc.; 10,000 negatives can be stored in the room. The portable outfit used in the wards has a 14-inch coil. It is connected by means of a length of cable upon a drum to the main supply from a wall plug.

Radiographic Room.

## Figures and Tables

**Figure f1:**
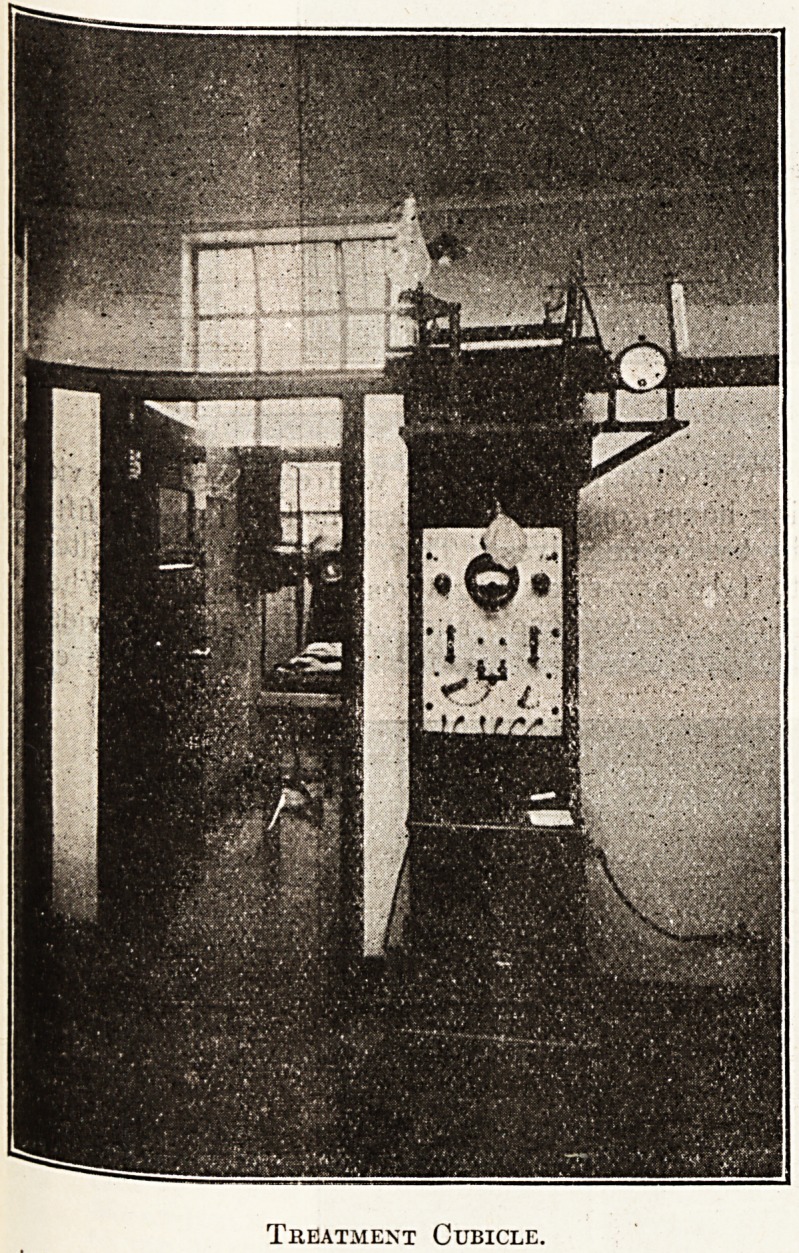


**Figure f2:**
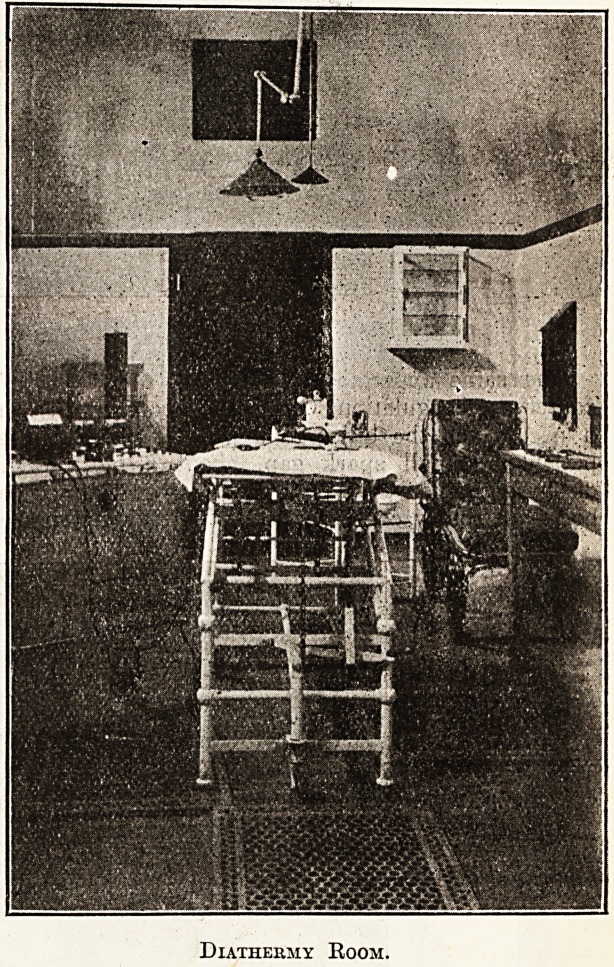


**Figure f3:**
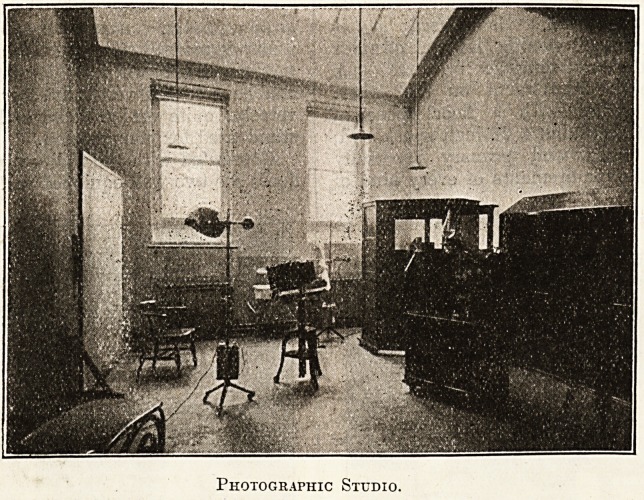


**Figure f4:**